# Predictive factors for effective selection of Interleukin-6 inhibitor and tumor necrosis factor inhibitor in the treatment of rheumatoid arthritis

**DOI:** 10.1038/s41598-020-73968-3

**Published:** 2020-10-06

**Authors:** Shinya Hayashi, Tsukasa Matsubara, Koji Fukuda, Keiko Funahashi, Marowa Hashimoto, Toshihisa Maeda, Tomoyuki Kamenaga, Yoshinori Takashima, Tomoyuki Matsumoto, Takahiro Niikura, Ryosuke Kuroda

**Affiliations:** 1 Department of Orthopaedic Surgery, Kobe University Graduate School of Medicine, 7-5-1 Kusunoki-cho, Chuo-ku, Kobe, 650-0017 Japan; 2 Department of Orthopaedic Surgery, Matsubara Mayflower Hospital, Kato, Japan; 3 Research Institute of Joint Diseases, Kobe, Japan

**Keywords:** Musculoskeletal system, Rheumatic diseases

## Abstract

Treatment of rheumatoid arthritis (RA) is aimed at long-term remission and inhibition of joint destruction by different biologic drugs. However, the choice of a particular biologic agent based on individual cases of RA remains unestablished. Interleukin-6 (IL-6) inhibitor and tumor necrosis factor (TNF) inhibitor are common biologics used for the treatment of RA. This study aimed to investigate predictive factors for effective selection of tocilizumab (IL-6 inhibitor) and etanercept (TNF inhibitor) in patients with RA. This is a retrospective cohort study. The 196 patients analyzed in this study were divided into four groups: tocilizumab treatment as the first biologic group (TCZ first, 42 patients), tocilizumab as second/ third biologic group (TCZ second, 34 patients), etanercept as the first biologic group (ETN first, 103 patients) and etanercept as second/third group (ETN second, 17 patients). Visual analog scale (VAS), clinical disease activity index (CDAI), and modified health assessment questionnaire (mHAQ) scores at the initiation of biologic treatment and after 6 months of tocilizumab and etanercept therapy were measured and compared to clinical parameters and radiographical parameters among the four groups. CRP, MMP-3, VAS, CDAI, and HAQ were improved after 6 months of treatment in all groups. Improvement of clinical outcomes was correlated with CRP value, duration of RA, and Sharp scores at the initiation of treatment. Multivariate analysis demonstrated improvement in CDAI was significantly associated with the yearly progression of erosion according to the Sharp score in TCZ first group (OR, 1.5; 95% CI, 1.03–2.07) and was negatively associated with the duration of RA (OR, 0.49; 95% CI, 0.29–0.86) at the initiation of treatment with ETN first group. We identified the predictive factors for effective selection of tocilizumab and etanercept treatment and established the effectiveness of tocilizumab for the patients with rapid progressive joint erosion and etanercept for the early administration from diagnosis of RA.

## Introduction

Rheumatoid arthritis (RA) is an autoimmune disease characterized by chronic inflammation of the synovial lining of the joint^1^, commonly observed with progressive joint destruction and systemic complications^2^. Joint inflammation causes bone erosions and cartilage loss, leading to the narrowing of the joint space, thereby resulting in joint deformities and functional impairments^2^. The treatment of RA has progressed with the introduction of biologic disease-modifying antirheumatic drugs (DMARDs) and standard implementation of the treat-to-target approach aimed at clinical remission, which has had a remarkable benefit in preventing joint damage and thereby improving the long-term outcomes in these patients^3^.

TNF plays a main role in RA, and many TNF inhibitors have been developed for the RA treatment^4–6^. TNF-α induces the secretion of multiple proinflammatory cytokines such as IL-1, -6, -8, granulocyte-macrophage colony-stimulating factor (GCS-F), and dickkopf (DKK)-1 which regulates Wnt pathway^7,8^. Previous reports showed that TNF inhibitor decreases serum DKK-1 levels in RA patients^8^, and DKK-1 levels correlated with destruction of radiographic change^9^. Therefore, the role of the Wnt pathway molecules including DKK-1 and sclerostin were important, however in vivo effect of TNF-α inhibitor on bone loss in RA patients are limited^10,11^.

IL-6, another pleiotropic cytokine with proinflammatory and immune stimulatory actions, plays a significant role in the pathogenesis of RA and disease activity^12^. The expression of serum IL-6 and IL-6 receptor is correlated with inflammation, clinical signs and symptoms, and radiographic signs of joint destruction^13,14^. IL-6 affects the function of neutrophils, T cells, B cells, monocytes, and osteoclasts that are highly activated in RA. IL-6 also affects the hepatic acute phase response, which is a key feature of RA^15^. As a key regulator in osteoclast differentiation, IL-6 may promote erosive joint changes by activating osteoclast formation and accelerating bone resorption^14^.

Etanercept is a fully human soluble TNF receptor Fc fusion protein that inhibits the binding of TNF receptors, blocking the proinflammatory activities of TNF receptor signaling^16^, and several studies demonstrated therapy with etanercept could inhibit the progression of joint destruction and repair bone erosion^17,18^. Tocilizumab is a humanized monoclonal antibody that inhibits the binding of IL-6 to the soluble- and membrane-expressed IL-6 receptors, blocking the proinflammatory activities of IL-6/IL-6 receptor signaling^19–21^. In a study involving patients with RA, tocilizumab monotherapy was found to be superior to conventional DMARDs for radiographic progression^22^. Similarly, tocilizumab treatment with methotrexate (MTX) considerably reduced cartilage degradation markers^26^.

Thus, rheumatologists can choose from several different types of biological DMARDs, including IL-6 and TNF inhibitors, for the treatment of RA. However, there are no established treatment guidelines for the selection of biologic drugs in individual patients based on their effectiveness. Tocilizumab and etanercept are commonly used as biological drugs for the treatment of RA^23,24^. Further, several reports showed the response to biological drugs was changed between the biologic treatment as the first drug and the treatment as the second drug because the immune response of the patient receiving second biologics may be altered after the first biological drug^25,26^. Therefore, the purpose of the present study was to evaluate the association between the patient factors and clinical outcome for the four groups (TCZ first, TCZ second, ETN first, and ETN second) to investigate the effective selection of biological drugs for the treatment of RA patients.

## Patients and methods

### Patient selection

This was a retrospective cohort study. Medical records of 196 patients with RA who were treated with tocilizumab at Matsubara Mayflower Hospital and Kobe University Hospital between October 2004 and September 2018 were analyzed. All patients included in the study were qualified according to the 1987 American College of Rheumatology RA criteria^27^. The inclusion criteria of this study were the patient who was treated with tocilizumab or etanercept for at least 6 months, and the administration of tocilizumab or etanercept treatment in the last 5 years of diagnosis as RA. The exclusion criteria of this study were the patient who was overlapped with tocilizumab and etanercept treatment. All the patients were treated with RA within three biological drugs. Finally, the 196 patients analyzed in this study were divided into four groups: tocilizumab treatment as the first biologic group (TCZ first, 42 patients), tocilizumab as second/third biologic group (TCZ second, 34 patients), etanercept as the first biologic group (ETN first, 103 patients) and etanercept as second/ third group (ETN second, 17 patients).

### Clinical evaluation

Collected data included the age, duration of RA, Steinbrocker class and stage, MTX dose (mg/week), glucocorticoid doses (mg/day), CRP, and MMP-3 at the time of introduction of the tocilizumab or etanercept therapy. The duration of RA was determined between the time of diagnosis as RA and the time of administration of tocilizumab or etanercept treatment. Data of modified HAQ (mHAQ), patients’ visual analog scale (VAS), and clinical disease activity index (CDAI) were obtained as clinical outcomes at the initiation of treatment and after 6 months of tocilizumab or etanercept therapy. Delta mHAQ, delta VAS, and delta CDAI were values of improvement between initiation of treatment and after 6 months of tocilizumab or etanercept therapy (values at the initiation of treatment-values at 6 M).

### Radiographic evaluation

Radiographs of the hands and feet were assessed according to the Sharp method^28^. All radiographic follow-up data included in the analysis were obtained within 5 years of the diagnosis of RA. In total, scores for the 196 radiographs (from 196 patients) were determined by a single experienced rheumatologist who was blinded to the clinical data. Sixteen and six areas were considered for assessing erosions and joint space narrowing (JSN) for the hands and feet, respectively. The maximum erosion score of the hands and wrists was 160, and that of the feet is 120 (maximum total erosion score: 280). Accordingly, the maximum JSN score of the hands and wrists was 120, and that of the feet is 48 (maximum total JSN score: 168). The sum of the erosion and JSN scores is the total Sharp/van der Heijde score (SHS) (maximum: 448). Therefore, radiographic joint destruction was quantified as the total SHS score divided by the duration of RA.

### Statistical analysis

Demographic and clinical characteristics of patients of TCZ first, TCZ second, ETN first, and ETN second with the statistical analysis are provided in Tables 1, 2, 3, 4 and 5. All data are expressed as mean ± standard deviation unless otherwise indicated. Patients' backgrounds were compared among four groups using one-way (Table 1) analysis of variance, with Tukey’s post hoc test for multiple comparisons of paired samples. To assess the improvement in laboratory data (CRP and MMP-3) and clinical scores (mHAQ, VAS, and CDAI), we compared values between initiation of treatment and 6 M using paired t-test (Table 2). Post-hoc power analysis was performed using G*Power 3^29^. For Pearson’s correlation analysis, the study is expected to provide a power (1 − β) of 0.87, 0.80, 0.99, and 0.55 for detecting an effect size q (H1) of 0.4, in TCZ first (n = 42), TCZ second (n = 34), ETN first (n = 103) and ETN second (n = 17) groups, respectively. In addition, for paired t-test, we calculated the effect size by means and SDs based on the Hedges’g for each parameter and the 95% confidence interval (CI) for effect sizes^30^.

**Table 1:** Patients background.

	Tocilizumab		Etanercept		*p*-value
	First	Second	First	Second	
Patient number	42	34	103	17	
Age	57.0 ± 15.9	57.9 ± 17.9	52.3 ± 13.0	51.9 ± 6.7	0.141
RA duration	1.8 ± 1.7	2.0 ± 1.9	2.2 ± 1.3	2.1 ± 1.2	0.266
Steinbrocker stage	2.5 ± 0.6	2.6 ± 1.1	2.4 ± 0.8	2.4 ± 0.9	0.642
Steinbrocker class	2.3 ± 0.9	2.0 ± 0.8	1.7 ± 0.6	1.4 ± 0.5	0.074
MTX dose (mg/week)	4.9 ± 3.9	3.1 ± 3.2	5.5 ± 4.4	6.2 ± 5.4	0.089
glucocorticoid dose (mg/day)	1.5 ± 3.8	3.7 ± 4.0	2.7 ± 2.8	3.9 ± 3.0	0.065
Total sharp score	71.4 ± 72.9	87.8 ± 91.4	45.0 ± 46.4	53.5 ± 63.6	**0.037**
Sharp score (erosion)	34.5 ± 40.1	42.3 ± 53.8	28.6 ± 34.9	25.9 ± 28.6	0.057
Sharp score (joint narrow)	37.6 ± 36.3	43.6 ± 44.3	25 ± 29.7	19.1 ± 18.6	**0.022**
Total sharp score/year	37.7 ± 45.8	50.6 ± 45.0	25.2 ± 27.1	22.4 ± 18.3	**0.001**
Sharp score (erosion)/year	21.2 ± 23.7	23.8 ± 24.0	13.1 ± 14.5	12.5 ± 10.9	0.057
Sharp score (joint narrow)/year	19.7 ± 21.8	26.8 ± 24.8	12.0 ± 14.0	9.8 ± 8.1	**0.001**

Values in bold font represent p-value less than 0.05.

**Table 2:** Comparison of laboratory data and clinical outcome between baseline and 6 months after drug administration in four groups.

	Baseline	6 M	*p*-value	Hedge’s g	95% CI	
**TCZ first**						
CRP	3.2 (2.9)	0.1 (0.5)	< 0.001	1.46	0.96	2.01
MMP-3	314 (230)	104 (85)	< 0.001	1.19	0.76	1.65
VAS	53 (34)	23 (24)	< 0.001	1.00	0.64	1.40
CDAI	34 (13)	13 (12)	< 0.001	1.64	1.20	2.15
Modified HAQ	4.3 (5.0)	2.1 (2.2)	0.004	0.56	0.21	0.93
**TCZ second**						
CRP	4.5 (3.5)	0.1 (0.4)	< 0.001	1.74	1.24	2.28
MMP-3	296 (207)	141(81)	< 0.001	0.97	0.61	1.35
VAS	56 (29)	30 (25)	< 0.001	0.94	0.63	1.28
CDAI	30 (15)	18 (12)	< 0.001	0.87	0.56	1.20
Modified HAQ	6 (5.9)	4.6 (3.3)	0.457	0.41	0.12	0.71
**ETN first**						
CRP	2.6 (3.3)	1.6 (0.7)	< 0.001	0.42	0.15	0.69
MMP-3	276 (262)	138 (138)	< 0.001	0.65	0.42	0.89
VAS	48 (27)	24 (25)	< 0.001	0.91	0.67	1.17
CDAI	23 (13)	7 (6)	< 0.001	1.57	1.24	1.91
modified HAQ	6.3 (2.5)	1.4 (2.5)	< 0.001	1.94	1.60	2.32
**ETN second**						
CRP	2.4 (2.0)	0.9 (0.9)	< 0.001	0.91	0.29	1.62
MMP-3	241 (244)	133 (104)	0.012	0.54	0.04	1.15
VAS	46 (37)	26 (30)	< 0.001	0.56	0.05	1.12
CDAI	20 (10)	8 (4)	< 0.001	1.49	0.74	2.38
Modified HAQ	4.3 (3.1)	1 (2.1)	0.021	1.20	0.56	1.96

Data represent as mean (standard deviation).

**Table 3:** Correlation clinical outcomes and clinical parameters at initiation of treatment.

	TCZ first			TCZ second			ETN first			ETN second		
	delta HAQ	delta VAS	delta CDAI	delta HAQ	delta VAS	delta CDAI	delta HAQ	delta VAS	delta CDAI	delta HAQ	delta VAS	delta CDAI
**CRP**												
rr	0.24	**0.40**	**0.44**	0.27	0.25	0.45	**0.36**	0.11	0.31	0.14	0.38	0.43
*p*-value	0.166	**0.017**	**0.016**	0.207	0.256	0.102	**0.007**	0.385	**0.038**	0.741	0.320	0.339
**Duration of RA**												
rr	0.12	0.13	0.12	0.15	0.26	0.01	0.03	**0.25**	**0.40**	0.34	**0.78**	**0.65**
*p*-value	0.534	0.465	0.534	0.472	0.264	0.994	0.798	**0.040**	**0.002**	0.196	**0.001**	**0.012**

Values in bold font represent p-value less than 0.05.

**Table 4:** Correlation clinical outcomes and radiographic parameters at initiation of treatment.

	TCZ first			TCZ second			ETN first			ETN second		
	delta HAQ	delta VAS	delta CDAI	delta HAQ	delta VAS	delta CDAI	delta HAQ	delta VAS	delta CDAI	delta HAQ	delta VAS	delta CDAI
**Total sharp score/year**												
rr	− 0.25	0.07	**0.45**	− 0.19	0.06	0.48	0.15	0.05	0.10	**0.66**	0.11	− 0.17
*p*-value	0.207	0.695	**0.033**	0.353	0.791	0.081	0.261	0.769	0.501	**0.005**	0.663	0.565
**Sharp score (erosion)/year**												
rr	− 0.21	0.07	**0.44**	− 0.27	0.13	**0.58**	0.13	0.05	0.09	**0.63**	− 0.03	− 0.13
*p*-value	0.291	0.723	**0.034**	0.193	0.595	**0.032**	0.311	0.696	0.562	**0.009**	0.908	0.826
**Sharp score (joint narrow)/year**												
rr	− 0.27	0.08	**0.43**	− 0.10	0.21	0.33	0.15	− 0.12	0.11	**0.56**	− 0.13	0.02
*p*-value	0.176	0.672	**0.041**	0.651	0.376	0.250	0.244	0.349	0.468	**0.025**	0.597	0.941

Values in bold font represent p-value less than 0.05.

**Table 5:** Results of the multivariate analysis for predictive factors of good response to treatment.

Drug	Predictive factor	Odds ratio (95% CI)	*p*-value
Tocilizumab first	Yearly progression of erosion Sharp score	1.5 (1.03–2.07)	0.033
Etanercept first	Duration of RA	0.49 (0.29–0.86)	0.012

Correlations between predictive factors (clinical and radiographic parameters) and clinical outcomes in each of the four groups were analyzed using Pearson’s correlation value (Tables 3, 4). To identify predictive factors of the effective selection of tocilizumab or etanercept therapy, we performed a multivariate analysis in the four groups to test the association among CRP, duration of RA, and radiographic parameters (TSS/year, Sharp score erosion/year, and Sharp score joint narrow/year) with the improvement in CDAI (Table 5). Improvement in CDAI was determined as following; patients were classified as remission (CDAI < 2.8), low activity (2.8 ≤ CDAI < 10), moderate activity (10 ≤ CDAI < 22), and high activity (CDAI ≥ 22) based on predefined cutoff values for CDAI, and we defined improvement of CDAI when disease activity class of the patient was changed to lower activity class after 6 months of tocilizumab or etanercept treatment. Predictive factors were identified using multivariate analysis. Odds ratios (ORs) and 95% confidence intervals (95% CIs) were calculated for multivariate analysis. Data were analyzed using SPSS version 19 J (IBM Japan, Tokyo, Japan).

### Ethics statement and patient consent

This study complies with the Declaration of Helsinki, and study protocols were approved by the ethics committee of the Research Institute of Joint Disease Kobe and Kobe University Graduate School of Medicine, and all participants provided informed consent for participation.

## Results

### Patient characteristics

The 196 patients analyzed in this study were divided into four groups. Patient characteristics of the four groups including age, duration of RA, Steinbrocker classification stage and class, weekly MTX and daily glucocorticoid dosages, and TSS including the Sharp score for erosion, Sharp for joint space narrowing, total Sharp score/year, sharp score for erosion/year, and Sharp score for joint space narrowing/year at initiation of biologics are provided in Table 1. One-way analysis of variance demonstrated significant differences of patient radiographic parameters of Total Sharp score, Sharp score for joint narrow, and sharp score for erosion/year were found among the four groups (Table 1). Post hoc analysis demonstrated significant differences and were found between TCZ first group and TCZ second group in Total Sharp score (*p* = 0.027), TCZ second group and ETN first in Sharp score for joint narrow (*p* = 0.022), and TCZ first and TCZ second in sharp score for erosion/year (*p* = 0.046), respectively.

### Biological drugs treatment improved laboratory data and clinical outcomes

Laboratory data and clinical outcomes of our study are provided in Table 2. Both tocilizumab and etanercept treatment for 6 months significantly improved laboratory data (CRP, MMP-3) and clinical outcomes (mHAQ, VAS, CDAI) except mHAQ in TCZ second group (Table 2).

### Improvement of clinical outcomes was correlated with CRP value and duration of RA at the initiation of treatment

The correlation between clinical outcomes and clinical parameters in tocilizumab or etanercept patients are provided in Table 3. There was no significant correlation between clinical parameters including age, Steinbrocker classification stage and class, weekly MTX dose, daily glucocorticoid dose, or MMP-3 and clinical outcomes (delta mHAQ, delta VAS, delta CDAI). However, there was a significant correlation between CRP value at initiation of treatment and the improvement of patients VAS or CDAI (delta VAS: rr = 0.40, *p* = 0.017; delta CDAI: rr = 0.44, *p* = 0.016) with tocilizumab first treatment and between CRP value and the improvement of patients mHAQ and CDAI (delta mHAQ: rr = 0.36, *p* = 0.007; delta CDAI: rr = 0.31, *p* = 0.038) with etanercept first treatment (Table 3). Further, significant correlation was found between RA duration and the improvement of patients VAS or CDAI (delta VAS: rr = 0.25, *p* = 0.040; delta CDAI: rr = 0.40, *p* = 0.002) with etanercept first treatment or the improvement of patients VAS or CDAI (delta VAS: rr = 0.78, *p* = 0.0001; delta CDAI: rr = 0.65, *p* = 0.012) with etanercept second/ third treatment (Table 3). Here, we demonstrated two clinical predictive factors (CRP, duration of RA) related to effective selection for tocilizumab and etanercept treatment by Pearson’s correlation analysis.

### Improvement of clinical outcomes was correlated with radiographic parameters at the initiation of treatment

The correlation between clinical outcomes and radiographic parameters, including yearly progression of TSS, erosion Sharp score, and joint narrow Sharp score, are shown in Table 4. A significant correlation between the yearly progression of erosion Sharp score and improvement in CDAI (delta CDAI) were found in TCZ first and second group (TCZ first: rr = 0.44, *p* = 0.034; TCZ second: rr = 0.58, *p* = 0.032) (Table 4). A significant correlation between improvement of HAQ (delta HAQ) and three radiographic parameters were found in ETN second group (TSS/year: rr = 0.66, *p* = 0.005, erosion Sharp score/year: rr = 0.63, *p* = 0.009, joint narrow Sharp score/year: rr = 0.56, *p* = 0.025). There was no significant correlation between clinical outcomes and radiographic parameters in ETN first group (Table 4). Here, we demonstrated three radiographic predictive factors (TSS/year, erosion Sharp score/year, joint narrow Sharp score/year) related to effective selection for tocilizumab and etanercept treatment by Pearson’s correlation analysis.

### Predictive factors of good response to tocilizumab and etanercept treatment

We demonstrated five significant predictive factors (CRP, duration of RA, TSS/year, erosion Sharp score/year, joint narrow Sharp score/year) related to effective selection for tocilizumab and etanercept treatment by Pearson’s correlation analysis. However, predictive factors might be dependent on multiple confounders. Therefore, these five significant predictive factors were used as covariates for multivariate analysis. We demonstrated that the improvement of CDAI was significantly associated with the yearly progression of erosion Sharp score (OR, 1.5; 95% CI, 1.03–2.07) at the initiation of treatment with TCZ first group and was negatively associated with duration of RA (OR, 0.49; 95% CI, 0.29–0.86) at the initiation of treatment with ETN first group (Table 5). There was no significant association between clinical factors or radiographic parameters and clinical outcomes in the patients with TCZ second group or ETN second group.

## Discussion

In the present study, we identified the predictive factors related to the effective selection of tocilizumab and etanercept treatment in RA. Improvement in CDAI was associated with the rapid progression of joint erosion before tocilizumab treatment as first biologic patients. The high correlation coefficients between improvement of CDAI and rapid progression of joint erosion was found in patients with tocilizumab treatment as second biologic, but the multivariate analysis did not show significant association. Recently, several reports demonstrated tocilizumab treatment to be more effective for the inhibition of bone erosion than inhibition of JSN^31–33^. Teitsma and colleagues reported that tocilizumab treatment with or without MTX in DMARD naïve patients with early RA is more effective in preventing progression of bone erosion compared to patients on MTX monotherapy; however, no significant difference in JSN was observed between the two groups^31^. FUNCTION study also showed that more progression of erosion was observed in patients with early RA on treatment with MTX alone compared to those treated with tocilizumab and MTX, but no significant difference in JSN was observed at 52 weeks^32,33^. From the point of cellular and molecular aspects, several studies demonstrated that the main activity of IL-6 on bone is its effect on osteoclastogenesis and bone resorption^34–36^. IL-6 stimulates osteoclast-like formation in long-term human marrow cultures by inducing IL-1 release^36^. IL-6 also mediates the stimulatory effects of TNF^37^ and enhances PTHrp-mediated hypercalcemia and bone resorption by increasing the pool of osteoclastic progenitors and their differentiation into mature osteoclasts^38^. TNF-a induces the production of RANKL both by indirectly influencing osteoclastogenesis supporting mesenchymal cells, and by directly influencing osteoclast precursor cells through the expression or the activation of factors such as RANK, TRAF6, and NF-kB, which are activated in the early phase of osteoclast differentiation^39^. However, the TNF-alpha may not directly induce osteoclastogenesis^38^, and TNF inhibitor therapy will not provide a complete reduction of bone damage in vivo study^40^. These studies supported our finding that improvement in CDAI was associated with the progression of joint erosion before initiation of tocilizumab treatment but not etanercept treatment.

We showed that the expression levels of MMP-3 was inhibited by tocilizumab and etanercept treatment in RA patients, and the expression levels at initiation of biologic treatment did not affect clinical outcomes between tocilizumab and etanercept treatment. Cartilage is mainly composed of collagen type II (70%) and proteoglycans, including aggrecan, and MMPs and aggrecanases are mediators of cartilage degradation^41^. MMP-3 is present in RA synovial fluid and overexpressed in rheumatoid synovium^37^. The expression levels of MMP-3 is associated with higher joint destruction in RA^38^. TNF blocker treatment decreased serum MMP-3 expression in RA patients^42^. We previously demonstrated that tocilizumab (IL-6 blocker) also inhibited serum MMP-3 expression through a similar mechanism as TNF inhibitor^43^. Therefore, the MMP-3 expression may not affect clinical outcomes between tocilizumab and etanercept treatment.

We also demonstrated that the duration of RA was negatively associated with improvement in CDAI in response to etanercept treatment as the first biologic. In other words, etanercept treatment is more effective for patients with early RA. Previous reports demonstrated aggressive treatment after the first 3 months of symptoms, with either conventional DMARD (csDMARD) or biologic DMARD (bDMARD) reducing the rate of disease progression^44^. Several studies attempt to evaluate the priority of bDMARD treatment as first-line for early RA patients^45–47^. However, bDMARD-naïve and MTX- naïve cohorts did not show a clear benefit of bDMARD as first-line^45–47^. Therefore, bDMARDs are still restricted to MTX- inadequate response, which avoids overtreatment. In our study, the average duration of RA in the etanercept first group was 1.8 years, and all the patients were treated with csDMARD prior to etanercept therapy. However, etanercept treatment was more effective for patients with shorter duration of RA. Therefore, we recommend the decision of switching from initial csDMARD to etanercept maybe earlier from initiation of RA treatment.

The limitation of this study is that the sample sizes in subgroups of the cohort were not large, and power is limited, especially in the ETN second group. Therefore, the data of ETN second has to be increased and re-analyzed in the future. Second, the patients’ radiographic background of Sharp score was different among the four groups. Sharp scores of tocilizumab as second/third biologic patients at initiation of treatment were higher than other groups because tocilizumab treatment was not recommended as a second biologic drug until EULAR 2013 recommendations presentation^48^. Although tocilizumab was used for more patients after the radiographic change, the study showed a significant correlation between improvement of CDAI and Sharp scores for erosion.

In conclusion, we identified the predictive factors related to the effective selection of tocilizumab and etanercept treatment. We propose the use of tocilizumab for the patients with rapid progressive joint erosion and etanercept for the early initiation of RA treatment.
